# Differences in Nursing Complexity and Intensity Across Stroke Subtypes: A Retrospective Study Using Standardized Nursing Language

**DOI:** 10.3390/brainsci16050471

**Published:** 2026-04-28

**Authors:** Manuele Cesare, Augusto Fusco, Gianfranco Damiani, Antonello Cocchieri

**Affiliations:** 1 A. Gemelli IRCCS University Hospital Foundation, 00168 Rome, Italy; antonello.cocchieri@policlinicogemelli.it; 2 Section of Hygiene, Department of Life Sciences and Public Health, Catholic University of the Sacred Heart, 00168 Rome, Italy; 3 UOSD High-Intensity Neurorehabilitation, Department of Neurosciences, Sensory Organs and Thorax, A. Gemelli IRCCS University Hospital Foundation, 00168 Rome, Italy; augusto.fusco@policlinicogemelli.it; 4 Hospital Hygiene Unit, A. Gemelli IRCCS University Hospital Foundation, 00168 Rome, Italy; gianfranco.damiani@policlinicogemelli.it

**Keywords:** stroke, ischemic stroke, hemorrhagic stroke, ischemic attack, transient, standardized nursing terminology, nursing diagnosis, nursing care, nursing informatics, neuroscience nursing

## Abstract

**Highlights:**

**What are the main findings?**

- Nursing complexity and nursing intensity did not vary in parallel across cerebrovascular conditions; ischemic and hemorrhagic strokes showed similar nursing complexity at admission, whereas TIA showed lower complexity.
- Nursing intensity followed a clearer gradient during hospitalization, being highest in hemorrhagic stroke, intermediate in ischemic stroke, and lowest in TIA.

**What are the implications of the main findings?**

- Standardized nursing language can make clinically meaningful differences in care demand more visible, particularly by distinguishing between early nursing needs identified at admission and the care provided throughout hospitalization.
- Capturing both nursing complexity and nursing intensity enables a more accurate and comprehensive representation of patient care requirements, strengthening the interpretation of care trajectories and supporting more informed clinical and organizational decision-making in stroke settings.

**Abstract:**

**Background/Objectives**: Ischemic stroke, hemorrhagic stroke, and transient ischemic attack (TIA) differ in terms of medical severity and prognosis; however, it remains unclear whether these differences are reflected in nursing complexity and nursing intensity when assessed using standardized nursing language. **Methods**: This retrospective study analyzed routinely collected nursing and administrative data from an acute care hospital. Hospitalizations were classified as ischemic stroke, hemorrhagic stroke, or TIA using ICD-9-CM codes. Nursing complexity was measured as the number of nursing diagnoses (NDs) documented within 24 h of admission, while nursing intensity was measured as the number of nursing actions (NAs) recorded during hospitalization. Group differences were tested using ANOVA and Kruskal–Wallis tests, as appropriate. **Results**: A total of 728 hospitalizations were included: 429 ischemic strokes, 236 hemorrhagic strokes, and 63 TIAs. Overall, 4136 NDs and 27,528 NAs were recorded. Distinct patterns emerged across stroke categories. ND counts differed significantly (F = 5.81, *p* = 0.003), with TIA showing lower counts than both ischemic and hemorrhagic stroke, while no significant difference was observed between ischemic and hemorrhagic stroke. NA counts also differed significantly (H = 16.73, *p* < 0.001), with the highest counts in hemorrhagic stroke, intermediate counts in ischemic stroke, and the lowest counts in TIA. In a sensitivity analysis standardized by length of stay, nursing intensity also differed significantly across stroke categories (H = 12.999, *p* = 0.002), although the pattern differed from that observed for cumulative counts. **Conclusions**: Nursing complexity and nursing intensity showed distinct patterns across stroke categories. While complexity was comparable between ischemic and hemorrhagic stroke and lower in TIA, intensity followed a clear gradient, highest in hemorrhagic stroke, intermediate in ischemic stroke, and lowest in TIA. Standardized nursing data may complement medical indicators by capturing additional dimensions of patient needs and care delivery in people with stroke.

## 1. Introduction

Stroke and related cerebrovascular events represent a leading cause of mortality, long-term disability, and healthcare utilization worldwide, posing substantial challenges to health systems and clinical practice [1,2]. In the acute and subacute phases, the care of patients with cerebrovascular events is particularly demanding from a nursing perspective, requiring continuous clinical surveillance, prevention of secondary complications, support for basic physiological functions, and close coordination within multidisciplinary teams. These care needs are dynamic and may evolve rapidly over time, reflecting not only neurological impairment but also functional, cognitive, and psychosocial changes [3,4].

Ischemic and hemorrhagic strokes have been extensively described and compared in terms of pathophysiology, clinical presentation, and outcomes [5,6]. Hemorrhagic stroke is generally associated with greater early clinical severity, hemodynamic instability, and higher medical resource consumption, including longer hospital stays and higher in-hospital mortality, compared to ischemic stroke [7,8]. In contrast, ischemic stroke spans a broader clinical spectrum and may involve multiple diagnostic and reperfusion pathways [9].

Transient ischemic attack (TIA), while traditionally considered a milder and transient cerebrovascular event, is increasingly recognized as part of the same disease continuum and as a marker of elevated short-term stroke risk, often requiring careful monitoring, secondary prevention, patient education, and structured follow-up [10]. Ischemic stroke, hemorrhagic stroke, and TIA together represent a heterogeneous group of cerebrovascular conditions that differ in medical and prognostic severity but share complex and evolving care needs [8,11].

From a nursing perspective, increasing attention has been devoted to the concept of complexity in stroke, particularly through approaches linking neurological severity and functional impairment to care dependency and staffing requirements [11]. Studies using patient classification systems have shown that greater clinical severity is generally associated with higher nursing workload, providing important evidence for workforce planning and resource allocation in specialized stroke settings [12]. However, such approaches often emphasize the intensity of care rather than the qualitative nature and diversity of nursing needs in everyday practice.

Nursing complexity can be conceptualized as a multidimensional construct reflecting the breadth, variability, and interrelatedness of patient care needs identified after the initial nursing assessment [13,14], whereas nursing intensity reflects the volume of care delivered in response to these needs over time. Operationally, these dimensions can be captured through standardized nursing diagnoses (NDs) identified at patient admission and nursing actions (NAs) recorded during the hospital stay [15,16]. In this context, standardized nursing language (SNL) provides a structured framework to quantify and compare nursing care, making clinical decision-making and care delivery analytically visible [17]. Unlike traditional workload or patient classification approaches, SNL enables a direct linkage between NDs and the corresponding NAs, thereby capturing the continuity between identified care needs and delivered care within routine clinical documentation.

Despite well-established differences between ischemic and hemorrhagic strokes in terms of medical severity and outcomes, it remains unclear whether these differences are reflected in nursing care profiles. This uncertainty is particularly relevant when TIA is considered within the same cerebrovascular spectrum, where clinical severity is lower, but care needs may still be substantial. Whether these conditions are associated with distinct patterns of nursing complexity and nursing intensity, when assessed through standardized nursing data, remains largely unexplored. Based on the existing literature, hemorrhagic stroke may be expected to involve greater nursing intensity during hospitalization because of its higher early clinical severity, instability, and supportive care needs, whereas TIA may be expected to show lower nursing complexity and intensity due to its generally milder presentation [3,4,7,10]. However, whether these differences in medical severity translate into distinct patterns of nursing complexity and nursing intensity, when assessed through SNL, remains uncertain.

Accordingly, the aim of the present study is to describe and compare both nursing complexity and nursing intensity among patients with ischemic stroke, hemorrhagic stroke, and TIA, using SNL to characterize early care needs (NDs) and care delivery patterns (NAs) across these cerebrovascular conditions.

## 2. Materials and Methods

### 2.1. Design

This study adopted a retrospective observational design based on routinely collected administrative data. The study was conducted and reported in accordance with the REporting of studies Conducted using Observational Routinely collected health Data (RECORD) reporting guidelines [18].

### 2.2. Setting

The study was conducted in an acute care hospital setting (1552 curative beds) that provides specialized care for patients with cerebrovascular conditions, located in Rome, Italy. The hospital hosts a dedicated Stroke Unit within the Neurology Department, equipped for the management of patients in the acute phase of strokes and ensuring continuous clinical and cardiorespiratory monitoring. The institution is integrated within the regional stroke network and operates as a referral hub, providing access to intravenous thrombolysis and endovascular thrombectomy for eligible patients. On-site neurosurgical operating facilities are available, allowing for the timely surgical management of hemorrhagic stroke when indicated. In addition, structured clinical care pathways for stroke care are in place, supporting multidisciplinary management across the continuum of care.

### 2.3. Inclusion Criteria

Patients were identified from the hospital administrative database and consecutively included, considering only the first eligible hospitalization per patient (index hospitalization) between 1 January and 31 December 2022, using a predefined set of ICD-9-CM diagnosis codes consistent with the American Heart Association/American Stroke Association classification proposed by Sacco et al. [19]. Admissions were included if associated with ICD-9-CM codes indicating central nervous system (CNS) infarction (433.01, 433.11, 433.21, 433.31, 433.81, 433.91, 434.01, 434.11, 434.91, 436, 336.1, 362.31, and 362.32), CNS hemorrhage (430, 431, and 432.9), or TIA (435.0, 435.1, 435.2, 435.3, 435.8, 435.9, and 362.34) [19].

### 2.4. Instruments for Data Collection and Study Variables

Data were obtained from routinely collected hospital information systems. Nursing-related data were extracted from the Professional Assessment Instrument (PAI), an electronic nursing documentation system supporting routine documentation of the nursing process. Within the PAI, NDs are coded using the Clinical Care Classification (CCC) System [20], whereas NAs are linked to the Italian Nomenclature of Nursing Care Performance (INNCP), providing a more granular description of bedside care activities through an automated background mapping process embedded in the system workflow [21]. The PAI incorporates a validated clinical decision support algorithm that generates evidence-informed suggestions of NDs and INNCP-coded NAs from nurse-entered assessment data, requiring active confirmation or rejection and thereby preserving clinical judgment [22]. Nurses working in the study setting receive ongoing annual training on the use of the PAI in accordance with Joint Commission International (JCI) quality and safety standards [23]. This training also includes support for diagnostic reasoning and the use of decision-support functionalities embedded in the documentation system, with the aim of promoting greater consistency in nursing assessment and recording practices.

In line with the conceptual framework adopted in this study, NDs documented within the first 24 h were considered indicators of nursing complexity, whereas NAs recorded throughout hospitalization were considered indicators of nursing intensity [14,15]. Nursing intensity was therefore operationalized as the cumulative volume of nursing care delivered during the hospital stay, rather than as a time-standardized daily rate.

Medical and administrative data were obtained from the Hospital Discharge Register (Scheda di Dimissione Ospedaliera, SDO), including sociodemographic (age, sex, and area of residence), organizational (admission modality), and clinical variables (stroke category, length of stay [LOS], and diagnosis-related group [DRG] weight as a proxy for medical complexity). Primary diagnosis codes were used to identify and classify hospitalizations into stroke categories.

Nursing and medical data were linked at the individual patient level using anonymized identifiers and analyzed to describe and compare nursing complexity and nursing intensity across cerebrovascular conditions.

### 2.5. Statistical Analyses

Descriptive statistics were used to summarize the demographic, clinical, and organizational characteristics of the study population. Continuous variables (e.g., age, DRG weight, LOS, number of NDs, and NAs) are reported as mean and standard deviation (SD) and as median and interquartile range (IQR) when appropriate. Categorical variables (e.g., sex, admission modality, and area of residence) are presented as frequencies and percentages. The distribution of continuous variables was assessed using skewness and kurtosis. Based on distributional properties, parametric or non-parametric tests were applied as appropriate.

Comparisons across stroke categories (ischemic stroke, hemorrhagic stroke, and TIA) were performed using one-way analysis of variance (ANOVA) for normally distributed variables, with post hoc pairwise comparisons conducted using Tukey’s Honest Significant Difference (HSD) test. For non-normally distributed variables, between-group differences were assessed using the Kruskal–Wallis H test, and the results are reported as mean ranks. As a sensitivity analysis, nursing intensity was also standardized by length of stay by calculating the average number of nursing actions per hospitalization day (NAs/LOS). This measure was intended as a pragmatic approximation of daily nursing intensity rather than a true time-resolved indicator.

The prevalence of NDs was calculated as absolute frequencies across the overall population and stratified by stroke category. NDs with a prevalence equal to or greater than 20% were classified as high-frequency NDs (HF-NDs) [24]. For descriptive clarity, only the ten most frequent NAs within each stroke category were reported individually, while all remaining actions were aggregated under the ‘Other’ category. Key descriptive results for nursing complexity and intensity were visually summarized using lollipop plots to facilitate the comparison of summary measures across stroke categories. No missing data were observed in the dataset; therefore, no imputation procedures were required.

All statistical analyses were performed using IBM SPSS Statistics^®^ for Mac OS, version 31.0.0.0 (IBM Corp., Armonk, NY, USA). Statistical significance was set at *p* < 0.05.

### 2.6. Ethical Considerations

The study was approved by the Ethics Committee of the Catholic University of the Sacred Heart, Rome, Italy (Protocol no. 0012915/24, ID 6752; 16 May 2024), and conducted in accordance with the Declaration of Helsinki, Good Clinical Practice, and applicable data protection regulations. Although retrospective and based on routinely collected data, study-specific informed consent was required under the approved protocol. During hospitalization, patients had provided consent for the processing of their personal data and for possible re-contact for research purposes; eligible participants were subsequently re-contacted and included only after providing study-specific consent. Data were extracted at the hospitalization level by the hospital ICT unit, de-identified, and analyzed in anonymized form. The study did not affect patient care.

## 3. Results

### 3.1. Study Population and Baseline Characteristics

A total of 728 hospitalizations that met the inclusion criteria were included in the analysis. Of these, 429 (58.9%) were classified as ischemic stroke, 236 (32.4%) as hemorrhagic stroke, and 63 (8.7%) as TIA. Overall, 408 patients were men (56.0%), with a comparable sex distribution across stroke categories. The mean age of the study population was 72.43 years (SD 14.26), with similar values across groups.

Most admissions occurred through the emergency department (94.8%), with proportions exceeding 90% for all stroke categories. Scheduled or other admissions accounted for a small proportion of cases (5.2%).

The mean DRG weight was 2.04 (SD 1.86), showing a gradient across stroke categories, with higher values in hemorrhagic stroke, intermediate values in ischemic stroke, and lower values in TIA. Similarly, the median length of stay (LOS) differed across groups, with hemorrhagic stroke showing the longest stays, ischemic stroke intermediate values, and TIA the shortest duration. The detailed characteristics stratified by stroke category are presented in Table 1.

### 3.2. Nursing Complexity and Nursing Intensity in the Study Population

Across the overall study population (N = 728), 4136 NDs and 27,528 NAs were recorded. The mean number of NDs per hospitalization was 5.68 (SD 3.36), with values ranging from 1 to 17. The median number of NAs per hospitalization was 22 (IQR 21), with values ranging from 1 to 728.

### 3.3. Nursing Complexity and Nursing Intensity Across Stroke Categories

When stratified by stroke category, nursing complexity and nursing intensity showed distinct patterns.

The mean number of NDs per hospitalization was comparable between ischemic stroke (5.73, SD 3.40) and hemorrhagic stroke (5.94, SD 3.49), while lower values were observed in TIA (4.35, SD 2.08).

**Figure 1:**
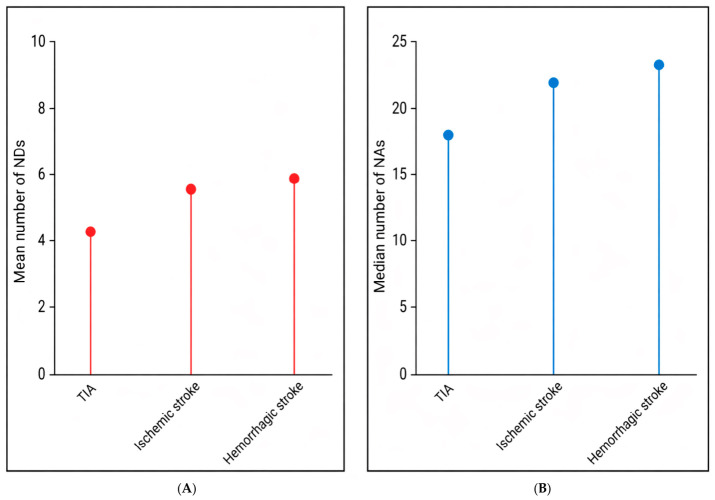
Lollipop plots of nursing complexity and nursing intensity across stroke categories. Legend: NDs, nursing diagnoses; NAs, nursing actions; TIA, transient ischemic attack. Note: (**A**) shows the mean number of nursing diagnoses (nursing complexity) per hospitalization across stroke categories, whereas (**B**) shows the median number of nursing actions (nursing intensity) per hospitalization. In both panels, the circular markers indicate the corresponding summary values (mean for NDs and median for NAs).

In contrast, nursing intensity followed a clearer gradient, with the median number of NAs highest in hemorrhagic stroke (24, IQR 28), intermediate in ischemic stroke (22, IQR 19), and lowest in TIA (18, IQR 9) (Figure 1).

### 3.4. Distribution of Nursing Diagnoses Across Stroke Categories

The prevalence of NDs showed a largely shared core profile across stroke categories. The risk of infection and the risk of falls were the most prevalent NDs in all three groups, followed by other recurrent care needs, such as *Acute pain*, *Impaired physical mobility*, *Sleep pattern disturbance*, and *Fluid volume deficit*. From a descriptive standpoint, hemorrhagic stroke appeared to show the broadest HF-ND profile, with a greater number of NDs exceeding the predefined 20% threshold compared with ischemic stroke and TIA. Some differences in ranking and prevalence nevertheless emerged across categories, particularly for hemorrhagic stroke and TIA. In particular, hemorrhagic stroke showed a broader profile of high-frequency NDs, whereas TIA was characterized by a narrower and more selective diagnostic profile. The detailed prevalence, percentage, and ranking of NDs across stroke categories are reported in the Supplementary Materials (Table S1).

### 3.5. Distribution of Nursing Actions Across Stroke Categories

The distribution of NAs showed a common core of recurring activities across stroke categories, mainly related to assessment, monitoring, safety, medication administration, and supportive care. However, differences in ranking and relative frequency emerged across groups, with hemorrhagic stroke showing a greater prominence of monitoring and supportive interventions and TIA a more focused action profile. These differences were reflected in the composition and ordering of the most frequent NAs across stroke categories. The detailed prevalence, percentage, and ranking of NAs across stroke categories are reported in the Supplementary Materials (Table S2).

### 3.6. Comparisons of Nursing Complexity and Nursing Intensity Across Stroke Categories

Comparisons across stroke categories showed statistically significant between-group differences for both NDs and NAs. For NDs, post hoc analyses indicated that TIA differed significantly from both ischemic and hemorrhagic stroke, whereas no significant difference emerged between ischemic and hemorrhagic strokes (ANOVA: F = 5.81, *p* = 0.003; Tukey HSD: TIA vs. ischemic mean difference = 1.38, *p* = 0.006; TIA vs. hemorrhagic mean difference = 1.60, *p* = 0.002; ischemic vs. hemorrhagic mean difference = −0.21, *p* = 0.711). For NAs, Kruskal–Wallis analysis also showed significant between-group differences, with the highest mean ranks in hemorrhagic stroke, followed by ischemic stroke and TIA (H = 16.73, df = 2, *p* < 0.001; mean ranks: hemorrhagic = 386.71, ischemic = 366.85, TIA = 265.26). Exploratory sex-stratified analyses showed statistically significant between-group differences among male patients but not among female patients, with broadly consistent directional patterns across stroke categories; these analyses are reported in the Supplementary Materials (Table S3).

### 3.7. Length-of-Stay-Standardized Nursing Intensity (Sensitivity Analysis)

For sensitivity analysis (see Section 2), nursing intensity was standardized by length of stay by calculating the average number of NAs per hospitalization day. This analysis confirmed significant differences across stroke categories (Kruskal–Wallis H = 12.999, df = 2, *p* = 0.002), with a distribution differing from that observed for cumulative counts: mean ranks were highest for TIA (409.63), intermediate for ischemic stroke (379.18), and lowest for hemorrhagic stroke (325.77).

## 4. Discussion

The present study compared nursing complexity and nursing intensity across ischemic stroke, hemorrhagic stroke, and TIA using standardized nursing data. The main finding was that these two dimensions did not vary in parallel across stroke categories. While nursing complexity was comparable between hemorrhagic and ischemic stroke and lower in TIA, nursing intensity followed a clearer gradient, with the highest values in hemorrhagic stroke, intermediate values in ischemic stroke, and the lowest in TIA. These findings suggest that medical severity and nursing care demand may overlap only partially. In particular, early nursing complexity did not appear to mirror differences in stroke subtype severity as clearly as the care delivered over the course of hospitalization.

This finding is partly consistent with previous studies showing that greater neurological severity and dependency are generally associated with higher nursing workload (i.e., NA counts) in stroke settings [11,12]. However, in our study, this pattern was more evident for nursing intensity than for nursing complexity at admission. Although hemorrhagic stroke was associated with greater clinical severity and longer hospitalization, the number of NDs identified within the first 24 h was comparable to that observed in ischemic stroke. This suggests that ND-based complexity captures a broader configuration of patient needs emerging from the initial nursing assessment, including functional, care-related, and safety dimensions, rather than acting as a direct proxy of stroke-specific neurological severity [15,25]. Accordingly, the similar ND counts observed between ischemic and hemorrhagic strokes should not be interpreted as evidence of equivalent clinical severity but rather as indicating that patients may present a comparably broad range of nursing-relevant problems at admission despite important differences in disease severity and subsequent clinical course. By contrast, the lower ND burden observed in TIA is coherent with the generally milder and more transient clinical presentation of these events [26,27].

Given these findings, the distinction between nursing complexity and nursing intensity should be framed according to their different temporal and conceptual roles. Nursing complexity captures the breadth of patient needs identified at admission, whereas nursing intensity reflects the volume of care delivered throughout hospitalization. Because these indicators are measured over different time windows, their divergence likely reflects both care demand and hospitalization dynamics. In particular, nursing intensity is influenced by clinical evolution, monitoring needs, supportive care, and length of stay [12,28]. This was further reflected in the sensitivity analysis standardized by LOS, in which the pattern across stroke categories differed from that observed for cumulative nursing intensity, suggesting that LOS contributed substantially to the cumulative gradient originally observed. Conversely, hemorrhagic stroke showed the longest LOS and the highest cumulative number of NAs. The descriptive distribution of NAs further clarifies this pattern: hemorrhagic stroke showed greater prominence of monitoring, skin care, repositioning, and specialized care activities, all of which are coherent with a more unstable and resource-intensive acute trajectory. Ischemic stroke, in turn, showed an intermediate intensity profile, indicating substantial care needs during hospitalization despite a generally less severe acute course. This pattern aligns with the literature, which identifies ischemic stroke as the largest contributor to stroke survivorship and long-term disability burden, with relevant ongoing care needs even when these are expressed differently from the more acute demands typical of hemorrhagic stroke [8].

A descriptive analysis of NDs and NAs further clarifies the nature of these differences. The ND profile showed a relevant shared core across stroke categories, with *Risk of infection* and *Risk of falls* as the most prevalent diagnoses in all groups, suggesting common nursing priorities in cerebrovascular care. Meanwhile, hemorrhagic stroke appeared to have a broader high-frequency ND profile, whereas TIA showed a narrower one. A similar pattern emerged for NAs: all groups shared a core of surveillance, safety, medication administration, and supportive care activities. However, hemorrhagic stroke was characterized by greater prominence of monitoring, skin care, and repositioning, whereas TIA showed a more focused action profile. Taken together, these findings indicate that stroke categories differ not only in the amount of care delivered but also in the composition of nursing care requirements according to clinical trajectory and care needs [3,29,30].

From a clinical and organizational perspective, these results may be relevant for staffing decisions, care planning, and the interpretation of nursing-sensitive indicators in stroke services. Patients may present a similarly broad set of nursing-relevant problems upon admission while requiring different levels and types of care during hospitalization, particularly in stroke settings characterized by rapidly evolving and heterogeneous trajectories [31].

This study also highlights the contribution of standardized nursing language in making these dimensions analytically visible. Although the specific configuration of the findings may depend in part on the documentation architecture adopted in the study setting, the broader value of structured nursing data in rendering care needs and care delivery analytically visible is likely relevant across different clinical contexts. By structuring both early care needs and recorded care activities, SNL enabled a more nuanced comparison of stroke populations beyond traditional clinical classifications alone. Previous evidence suggests that nursing data can enhance outcome models and improve the representation of patient care [16,17]. In this context, the use of SNL is relevant not only for describing nursing care more accurately and strengthening its visibility in clinical and organizational analyses but also for supporting the study of how nursing problems identified at admission and care delivered during hospitalization may shape patient outcomes.

Some limitations should be acknowledged. First, the retrospective design based on routinely collected data may have introduced information bias, although no missing data were observed in the study variables. Second, nursing documentation may vary across professionals, even though nurses in the study setting receive ongoing training in the use of the documentation system, including diagnostic reasoning and decision-support functionalities embedded in the PAI. However, this training may reduce, but not fully eliminate, variability in documentation practices [32]. Moreover, because nurse-level identifiers were not available, inter-nurse variability in documentation could not be directly assessed or statistically controlled. Accordingly, the measures of nursing complexity and nursing intensity adopted in this study may reflect not only patient care needs and care delivery but also documentation practices and workflow variability. Third, according to our framework, nursing complexity and nursing intensity were measured over different time windows. This methodological asymmetry may have contributed, at least in part, to the divergent patterns observed across stroke categories and warrants cautious interpretation of the comparison between these two dimensions. In addition, the study groups were unbalanced in size, particularly with regard to the relatively small TIA subgroup. Although this distribution likely reflects real-world hospitalization patterns in a tertiary stroke care setting, it may have reduced statistical power for some intergroup comparisons and limited the stability of subgroup-specific estimates. In addition, because nursing intensity was measured as the total number of NAs recorded during hospitalization, it was likely influenced by differences in length of stay across stroke categories. Although day-level granularity of NAs was not available, a sensitivity analysis standardized by LOS was performed using the average number of NAs per hospitalization day. However, this measure should be interpreted as a crude LOS-standardized estimate rather than a true time-resolved measure of nursing intensity, and therefore, it cannot fully disentangle daily care demand from hospitalization dynamics. Moreover, because stroke-specific severity measures (e.g., NIH Stroke Scale—NIHSS) were not available, we could not fully disentangle whether the higher cumulative nursing intensity observed in hemorrhagic stroke was mainly related to greater clinical severity or longer hospitalization. An additional limitation concerns the use of ICD-9-CM codes for the identification and classification of cerebrovascular conditions. Although widely adopted in administrative datasets, these codes may not fully capture the clinical complexity and heterogeneity of stroke presentations, particularly when relying on primary diagnoses alone. The potential underrepresentation of secondary conditions may have influenced the characterization of patient profiles and, consequently, the interpretation of nursing complexity and intensity. Furthermore, the study was conducted in a single tertiary referral center operating as a regional hub for stroke care. This organizational model is likely to concentrate patients with higher clinical severity and complexity, which may limit the generalizability of the findings to settings with lower levels of care intensity, such as spoke hospitals or non-specialized units. Finally, the study was conducted in a single center using one documentation system, which may limit the generalizability of the findings to settings with different organizational models or nursing information systems. This is particularly relevant for contexts in which nursing documentation is less structured or relies on different terminologies, as the observed distributions of nursing diagnoses and nursing actions may be partly influenced by the degree of standardization embedded in local documentation practices.

Despite these limitations, this study provides novel evidence by jointly examining nursing complexity and nursing intensity in patients with ischemic stroke, hemorrhagic stroke, and TIA using standardized nursing data derived from routine practice. Further studies should validate these findings in multicenter settings, explore their relationship with patient outcomes, and assess whether integrating nursing-sensitive data with medical indicators improves the representation of patient complexity. In addition, linking standardized nursing measures derived from the PAI with established stroke-specific severity and functional scales (e.g., NIHSS or dependency measures) may help clarify the extent to which these approaches capture overlapping or distinct dimensions of patient condition. The availability of time-resolved nursing data would allow a more refined standardization of nursing intensity and a more accurate assessment of how LOS and daily care demands jointly shape differences across stroke categories. Moreover, examining potential sex-related differences in nursing complexity and nursing intensity across stroke categories may provide additional insights into care needs and their variability. Finally, extending the analysis across the care pathway using standardized nursing data may provide a more comprehensive understanding of care trajectories and their relationship with patient outcomes. Such approaches could further support the development of integrated models combining medical and nursing data to better represent patient complexity in clinical and organizational decision-making.

## 5. Conclusions

This study shows that nursing complexity and nursing intensity do not vary in parallel across ischemic stroke, hemorrhagic stroke, and TIA. While early nursing complexity was similar in ischemic and hemorrhagic stroke and lower in TIA, nursing intensity followed a clearer gradient, with the highest values in hemorrhagic stroke, intermediate values in ischemic stroke, and the lowest in TIA. These findings support the distinction between patient care needs identified at admission and the volume of care delivered during hospitalization while indicating that cumulative differences in nursing intensity should be interpreted in light of LOS. They also highlight the contribution of standardized nursing language in making nursing care visible and analytically comparable, thereby supporting more comprehensive clinical and organizational interpretations of stroke care.

## Figures and Tables

**Table 1:** Patient characteristics stratified by stroke category.

Variable	Total (N = 728)	Ischemic (n = 429)	Hemorrhagic (n = 236)	TIA (n = 63)
Age, mean (SD)	72.43 (14.26)	73.11 (13.94)	71.57 (15.09)	71.06 (13.10)
Male, n (%)	408 (56.0)	242 (56.4)	129 (54.7)	37 (58.7)
Female, n (%)	320 (44.0)	187 (43.6)	107 (45.3)	26 (41.3)
Admission from ED, n (%)	690 (94.8)	410 (95.6)	221 (93.6)	59 (93.7)
Scheduled/other, n (%)	38 (5.2)	19 (4.4)	15 (6.4)	4 (6.3)
DRG weight, mean (SD)	2.035 (1.8607)	1.9717 (1.5227)	2.4719 (2.4187)	0.8302 (0.4323)
LOS, median (IQR)	7.00 (9)	7.00 (8)	10.00 (17)	5.00 (4)

Legend: TIA, transient ischemic attack; SD, standard deviation; ED, emergency department; DRG, diagnosis-related group; LOS, length of stay; IQR, interquartile range.

## Data Availability

The data presented in this study are available upon request from the corresponding author due to privacy, legal, and ethical restrictions governing the use of routinely collected clinical data.

## Supplementary Materials

The following supporting information can be downloaded at: https://www.mdpi.com/article/10.3390/brainsci16050471/s1, Table S1. Prevalence, percentage, and ranking of nursing diagnoses across stroke categories. Table S2. Prevalence, percentage, and ranking of nursing actions across stroke categories. Table S3. Exploratory sex-stratified analyses of nursing complexity and nursing intensity across stroke categories.

## Abbreviations

The following abbreviations are used in this manuscript:

ANOVA	Analysis of Variance
CCC	Clinical Care Classification
CNS	Central Nervous System
DRG	Diagnosis-Related Group
ED	Emergency Department
HF-NDs	High-Frequency Nursing Diagnoses
HSD	Honest Significant Difference
ICD-9-CM	International Classification of Diseases, Ninth Revision, Clinical Modification
ICT	Information and Communication Technology
INNCP	Italian Nomenclature of Nursing Care Performance
IQR	Interquartile Range
JCI	Joint Commission International
LOS	Length of Stay
NAs	Nursing Actions
NDs	Nursing Diagnoses
NIHSS	National Institutes of Health Stroke Scale
PAI	Professional Assessment Instrument
RECORD	REporting of studies Conducted using Observational Routinely-collected health Data
SD	Standard Deviation
SDO	Scheda di Dimissione Ospedaliera
SNL	Standardized Nursing Language
TIA	Transient Ischemic Attack
