# Supplementary material for: Differences in Nursing Complexity and Intensity Across Stroke Subtypes: A Retrospective Study Using Standardized Nursing Language
Source: Brain Sci. 2026 Apr 28;16(5):471. doi: 10.3390/brainsci16050471 (PMC13204901; doi:10.3390/brainsci16050471)
Supplement: Supplementary file 1 [file brainsci-16-00471-s001.zip › brainsci-4269227-supplementary.pdf]

Table S1. Prevalence, percentage, and ranking of nursing diagnoses across stroke categories

ND	General population (N=4,136) n (%)	Rank	Ischemic stroke (N=2,459) n (%)	Rank	Hemorrhagic stroke (N=1,403) n (%)	Rank	TIA (N=274) n (%)	Rank
Risk of infection	486 (**66.8**)	1	275 (**64.1**)	1	168 (**71.2**)	1	43 (**68.3**)	1
Risk of falls	426 (**58.5**)	2	248 (**57.8**)	2	142 (**60.2**)	2	36 (**57.1**)	2
Acute pain	320 (**44.0**)	3	198 (**46.2**)	4	97 (**41.1**)	4	25 (**39.7**)	4
Impaired physical mobility	313 (**43.0**)	4	188 (**43.8**)	6	107 (**45.3**)	3	18 (**28.6**)	6
Sleep pattern disturbance	311 (**42.7**)	5	211 (**49.2**)	3	66 (**28.0**)	8	34 (**54.0**)	3
Fluid volume deficit	284 (**39.0**)	6	193 (**45.0**)	5	70 (**29.7**)	7	21 (**33.3**)	5
Risk for impaired skin integrity	265 (**36.4**)	7	155 (**36.1**)	7	95 (**40.3**)	5	15 (**23.8**)	7
Risk of injury	256 (**35.2**)	8	153 (**35.7**)	8	91 (**38.6**)	6	12 (19.0)	9
Imbalanced nutrition	208 (**28.6**)	9	112 (**26.1**)	9	85 (**36.0**)	10	11 (17.5)	10
Bathing self-care deficit	149 (**20.5**)	10	89 (**20.7**)	10	57 (**24.2**)	–	3 (4.8)	–
Urinary elimination alteration	145 (19.9)	–	82 (19.1)	–	50 (**21.2**)	–	13 (**20.6**)	8
Aspiration risk	113 (15.5)	–	54 (12.6)	–	58 (**24.6**)	9	1 (1.6)	–
Toileting self-care deficit	98 (13.5)	–	61 (14.2)	–	35 (14.8)	–	2 (3.2)	–
Activity intolerance risk	95 (13.1)	–	64 (14.9)	–	25 (10.6)	–	6 (9.5)	–
Swallowing impairment	86 (11.8)	–	47 (11.0)	–	37 (15.7)	–	2 (3.2)	–
Ineffective breathing pattern	84 (11.5)	–	45 (10.5)	–	35 (14.8)	–	4 (6.3)	–
Activity intolerance	81 (11.1)	–	45 (10.5)	–	27 (11.4)	–	9 (14.3)	–
Feeding self-care deficit	77 (10.6)	–	43 (10.0)	–	31 (13.1)	–	3 (4.8)	–
Dressing/grooming deficit	62 (8.5)	–	43 (10.0)	–	19 (8.1)	–	0 (0.0)	–
Impaired skin integrity	62 (8.5)	–	37 (8.6)	–	24 (10.2)	–	1 (1.6)	–
Constipation	53 (7.3)	–	30 (7.0)	–	14 (5.9)	–	9 (14.3)	–
Anxiety	45 (6.2)	–	25 (5.8)	–	18 (7.6)	–	2 (3.2)	–
Disturbed body image	23 (3.2)	–	5 (1.2)	–	17 (7.2)	–	1 (1.6)	–
Acute confusion	19 (2.6)	–	12 (2.8)	–	7 (3.0)	–	0 (0.0)	–
Impaired tissue perfusion	17 (2.3)	–	9 (2.1)	–	8 (3.4)	–	0 (0.0)	–
Fatigue	14 (1.9)	–	10 (2.3)	–	4 (1.7)	–	0 (0.0)	–
Noncompliance	11 (1.5)	–	8 (1.9)	–	2 (0.8)	–	1 (1.6)	–
Bowel incontinence	10 (1.4)	–	6 (1.4)	–	4 (1.7)	–	0 (0.0)	–
Diarrhea	9 (1.2)	–	4 (0.9)	–	3 (1.3)	–	2 (3.2)	–

Chronic pain	7 (1.0)	–	3 (0.7)	–	4 (1.7)	–	0 (0.0)	–
Ineffective coping	3 (0.4)	–	1 (0.2)	–	2 (0.8)	–	0 (0.0)	–
Fear	3 (0.4)	–	3 (0.7)	–	0 (0.0)	–	0 (0.0)	–
Social interaction impairment	1 (0.1)	–	0 (0.0)	–	1 (0.4)	–	0 (0.0)	–

Legend: NDs, nursing diagnosis; TIA, transient ischemic attack; n, absolute frequency; %, percentage calculated on the total number of patients in each group.

Note: Rank, position of each nursing diagnosis based on its frequency within the corresponding population. High-frequency nursing diagnoses (≥20%) are indicated by bold percentages within each corresponding category.

Table S2. Prevalence, percentage, and ranking of nursing actions across stroke categories

INNCP-coded NAs	General population (N=27,528) n (%)	Rank	Ischemic stroke (N=15,927) n (%)	Rank	Hemorrhagic stroke (N=10,350) n (%)	Rank	TIA (N=1,251) n (%)	Rank
Assessment of the patient's dependency levels through physical examination (A.01.01)	985 (3.6)	1	529	1	402	1	54	4
Administration of prescribed medications via the enteral route (A.07.01)	747 (2.7)	2	473	3	218	8	56	3
Assessment of the duration and quality of daytime and nighttime rest and sleep, including factors affecting them (A.16.01)	746 (2.7)	3	488	2	–	–	66	1
Application of an identification wristband to the patient (A.20.06)	741 (2.7)	4	464	4	229	7	48	6
Provision of devices to reduce hazards (e.g., side rails, floor mats, handrails, non-slip	713 (2.6)	5	408	6	269	4	36	9

surfaces) (A.20.04)								
Management of peripheral venous catheter (e.g., site care, dressing change, maintenance of patency) (A.05.04)	672 (2.4)	6	454	5	–	–	50	5
Monitoring and recording of the patient’s diet, food balance, and hydration status (A.12.05)	671 (2.4)	7	406	7	–	–	61	2
Skin monitoring (A.19.16)	665 (2.4)	8	–	–	296	2	–	–
Complete bed bath (A.19.07)	639 (2.3)	9	–	–	276	3	–	–
Objective assessment of the patient’s risk of injury/falls (e.g., Conley scale) (A.20.03)	615 (2.2)	10	391	8	–	–	–	–
Environmental assessment, hazard mapping, and proposals for changes to ensure comfort and safety (A.20.01)	–	–	377	9	–	–	38	8
Management of intravenous infusion (e.g.,	–	–	374	10	–	–	39	7

monitoring flow rate, allergic reactions, insertion site) (A.07.05)								
Use of devices to reduce skin pressure (A.19.15)	–	–	–	–	245	5	–	–
Monitoring of level of consciousness (A.03.02)	–	–	–	–	238	6	–	–
Preparation of the patient for a diagnostic examination (A.06.02)	–	–	–	–	212	9	38	8
Collaboration with the physician in a specialized setting (A.10.01)	–	–	–	–	210	10	–	–
Repositioning of the patient every 1–2 hours (A.15.08)	–	–	–	–	210	10	–	–
Administration of prescribed medications via the parenteral route (A.07.02)	–	–	–	–	–	–	38	8
Measurement of one or more vital signs (A.02.01)	–	–	–	–	–	–	33	10
Identification of known allergies (e.g., drugs, foods, insects,	–	–	–	–	–	–	33	10

environmental allergens) and usual reactions (A.20.02)				
Other	20,334 (73.9)	11,563 (72.4)	7,545 (72.9)	661 (52.8)

Legend: INNCP, Italian Nomenclature of Nursing Care Performance; NAs, nursing actions; TIA, transient ischemic attack; n, absolute frequency; %, percentage calculated on the total number of nursing actions within each group.
Note: Rank indicates the position of each nursing action based on its frequency within the corresponding population. For each group, only the ten most frequent nursing actions within each group are reported individually; actions not included in the top ten for a given group are indicated with a dash (–) and are included in the 'Other' category.

Table S3. Exploratory sex-stratified analyses of nursing complexity and nursing intensity across stroke categories.

Variable	Ischemic stroke, male (n=242)	Ischemic stroke, female (n=187)	Hemorrhagic stroke, male (n=129)	Hemorrhagic stroke, female (n=107)	TIA, male (n=37)	TIA, female (n=26)	Overall test
NDs, mean (SD)	5.65 (3.60)	5.84 (3.13)	5.70 (3.37)	6.24 (3.62)	4.00 (2.13)	4.85 (1.93)	Male: $F = 4.003^{*}$, $p = 0.019$; Female: $F = 2.022^{*}$, $p = 0.134$
NAs, median (IQR)	21.00 (18)	24.00 (23)	25.00 (27)	24.00 (38)	16.00 (8)	21.00 (13)	Male: $H = 13.514^{\dagger}$, $p = 0.001$; Female: $H = 4.415^{\dagger}$, $p = 0.110$

Legend: TIA, transient ischemic attack; NDs, nursing diagnoses; SD, standard deviation; NAs, nursing actions; IQR, interquartile range. Note: *ANOVA test; †Kruskal–Wallis test. Overall tests refer to comparisons across stroke categories within each sex.
